# Building and revolutionising public healthcare: A living ecosystem to link and improve patient health data and outcomes in a Brazilian hospital

**DOI:** 10.23889/ijpds.v8i2.2352

**Published:** 2023-09-14

**Authors:** Vilson Cobello Junior, Antonio Jose Rodrigues Pereira, Francis Mironescu Tomazini, Gisele Regina Pereira da Silva, Alcir Alves dos Santos Junior, Claudio Tadashi Fukuda, Davi Rocha da Silvia, Gladis Aparecida de Faria, Djavan Costa Nonato, Evelinda Trindade, Geraldo Busatto Filho

**Affiliations:** 1 Clinicas Hospital of Sao Paulo University Medicine School, Sao Paulo, Brazil

## Objectives

To develop a Brazilian public hospital, Sao Paulo University Medical School Clinics Hospital, HCFMUSP, informational model to link and improve multiple patients' health data, care pathways and outcomes, to build a living real world ecosystem aiming to subsidize policy decision-making, support research and promote patients' engagement and involvement.

## Methods

Policy-relevant linkage data including demography, diagnostics, outpatient and emergency room visits, hospitalizations, intensive care evolution, assisted mechanical ventilation or special equipment’s uses, electronic prescriptions, imaging and clinical laboratory tests results, surgery records, blood components use, and medical and multidisciplinary teams’ evolutions. Telemedicine-based hub developed for patient’s access to his own visits or procedures schedule, comprehensive data and results temporal series, and specific communications channels. Anonymized data sharing for Sao Paulo State Health Secretariat policy decision-making and SP Research Agency multicenter Data Lake for Covid-19 pandemic research. Stratified impact and economic analysis regarding clinical and co-morbid conditions research were published.

## Results

Since March 2020, this informational model example comprises over 10,000 Covid-19 patient’s related data with more than 100,000 events registered. During the first pandemic trimester, upon SP Health Secretariat policy, the HCFMUSP Central Institute’s 900 ward and 300 ICU beds were the SP central reference for severe and critical admissions. In this first evaluation 88.4% had co-morbidities (e.g. 48.1% hypertension, 30.5% diabetes), 51.7% required ICU admission and 28.9% died. Average hospital length of stay was 10.7 days, mean cost per admission was US$12,637.42, and the overall daily cost was US$919.24. Age strata >69 years confirmed COVID-19, ICU, elevated C-reactive protein (inflammation) adjusted by D-dimer levels (thrombosis biomarker), higher mSOFA, mechanical ventilation, dialysis, surgery and comorbidities, remained significantly associated with higher (24%-200%) costs and poorer outcomes.

## Conclusion

The informational model is proving to be beneficial for all stakeholders. Technology-based organized systems increased management accuracy and efficiency, emergency preparedness, facilitates patient’s involvement and participation, promote medical and multi-professionals teams’ knowledge development, and permits to subsidize policy decisions and to improve public health.

